# Le syndrome de la pince aorto-mésentérique chez l'enfant: à propos d'un cas primitive

**DOI:** 10.11604/pamj.2014.19.151.4651

**Published:** 2014-10-15

**Authors:** Mbaye Fall, Papa Abdoulaye Bâ, Fodé Baba Touré, Pape Alassane Mbaye, Gabriel Ngom

**Affiliations:** 1 Hôpital de Pikine, Dakar, Sénégal; 2 Unité de Formation et de Recherche des Sciences de la Santé, Université de Thiès, Thiès, Sénégal; 3 Hôpital Aristide Le Dantec, Dakar, Sénégal

**Keywords:** Syndrome de la pince aorto-mésentérique, duodéno-jéjunostomie, artère mésentérique supérieure, enfant, Aorto-mesenteric compression syndrome, duodeno-jejunostomy, superior mesenteric artery, child

## Abstract

Le syndrome de la pince aorto-mésentérique résulte de la compression du troisième duodénum entre l'artère mésentérique supérieure et l'aorte. Elle détermine un tableau d'occlusion intestinale haute aiguë ou chronique. Nous faisons une revue de la littérature à partir d'un cas de syndrome de la pince aorto-mésentérique. Il s'agit d'un nourrisson de 16 mois suivi pour des vomissements alimentaires évoluant depuis l’âge de 03 mois et rebelles au traitement médical. Il s'agit de vomissements postprandiaux tardifs d'apparition intermittente, améliorés par la position de décubitus ventral. Le bilan radiologique composé d'une radiographie de l'abdomen sans préparation, d'un transit oeso-gastroduodénal (TOGD) et d'un angioscanner a permis d'aboutir au diagnostic d'obstruction extrinsèque et incomplète du 3ème duodénum par la pince aorto-mésentérique. Une dérivation interne par duodéno-jéjunostomie sur anse en oméga a permis de contourner l'obstacle vasculaire avec des suites simples. Le TOGD de contrôle du 9ème jour postopératoire a montré une bonne perméabilité de l'anastomose duodéno-jéjunale. Les vomissements ont disparu et on note un gain pondéral de 2 kg en 3 mois. Les vomissements chroniques chez le nourrisson sont d’étiologies variées. L'obstacle siégeant au 3ème duodénum est le plus souvent dû à une pince aorto-mésentérique. Malheureusement ce diagnostic est rarement porté surtout en Afrique probablement du fait de l'insuffisance des moyens diagnostiques en particulier dans les zones reculées. Le traitement peut être fait par les moyens chirurgicaux ou nutritionnels.

## Introduction

Le syndrome de la pince mésentérique se définit par la compression de la troisième portion du duodénum (D3) entre l'artère mésentérique supérieure (AMS) en avant et le plan aorto-rachidien en arrière [1–3]. Il s'agit d'une affection multifactorielle, rare chez l'enfant. Un amaigrissement important, une déformation du rachis lombaire et des anomalies anatomiques (un ligament de Treitz court, une insertion basse de l'artère mésentérique supérieure sur l'aorte) sont les facteurs favorisants les plus fréquemment retrouvés [3, 4]. Nous rapportons un cas primitif de syndrome de la pince aorto-mésentérique observé chez un nourrisson et discutons les aspects étiopathogéniques, les moyens diagnostiques et thérapeutiques.

## Patient et observation

CAT Sall, est un nourrisson de 16 mois, issu d'un mariage consanguin du 2éme degré. Il a été adressé dans notre service pour des vomissements alimentaires postprandiaux tardifs évoluant depuis l’âge de 3 mois. Il avait été opéré à l’âge de 10 mois pour des végétations adénoïdes. A l'examen clinique, il était en assez bon état général et pesait 9,2 Kg pour une taille de 81 cm; l’état nutritionnel était satisfaisant et il n'existait pas de pli de déshydratation. Son abdomen était souple indolore sans masse palpable ni ondulations péristaltiques visibles, mais légèrement ballonné. La biologie révélait une anémie normochromenormocytaire. L'ionogramme sanguin retrouvait une hyponatrémie à 128 mmol/l, une hypokaliémie à 3,1 mmol/l et une hypochlorémie à 85 mmol/l.

**Figure 1:**
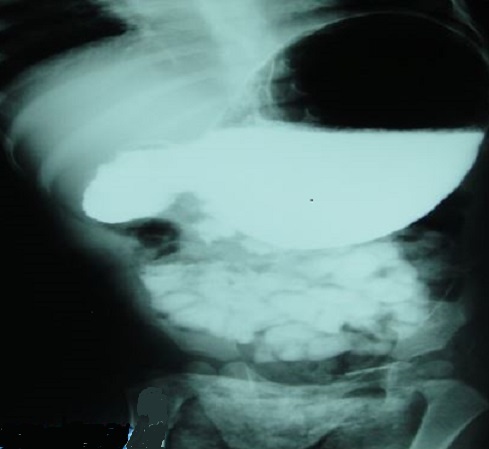
Transit gastroduodénal: dilatation importante de l'estomac et du deuxième duodénum en amont de la sténose extrinsèque

**Figure 2:**
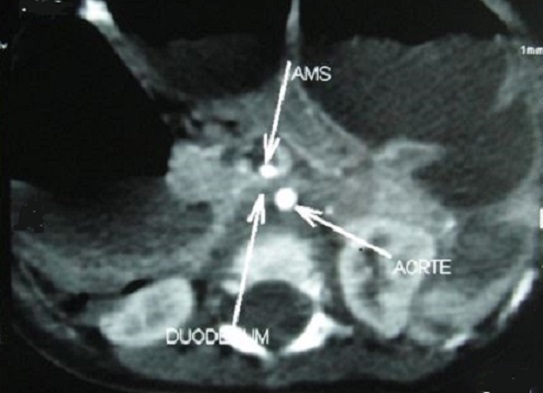
Coupe transversale TDM passant par la troisième portion duodénale révélant une compression du troisième duodénum par l'artère mésentérique supérieure (AMS) sur l'aorte

**Figure 3:**
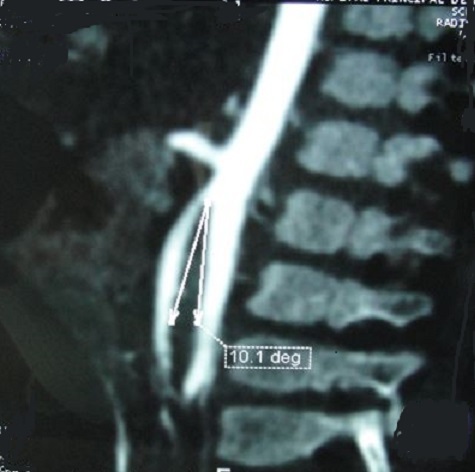
Angio-scanner aorto-mésentérique : l'angle entre l'aorte et l'artère mésentérique supérieure est aigu, mesuré à 10

La radiographie de l'abdomen sans préparation montrait une volumineuse clarté dessinant les contours de l'estomac avec présence d'un niveau hydro-aérique en regard du flanc droit de la 12^éme^vertèbre dorsale. Le transit gastroduodénal à la gastrografine mettait en évidence une dilatation de l'estomac, des premier et second duodénums, de la portion proximale de D3 avec arrêt de la progression du produit de contraste évoquant une sténose duodénale (Figure 1). L'angio-scanner confirmait la compression extrinsèque du 3^éme^ duodénum sous l'angle aorto-mésentérique mesuré à 10° (Figure 2 et Figure 3).

**Figure 4:**
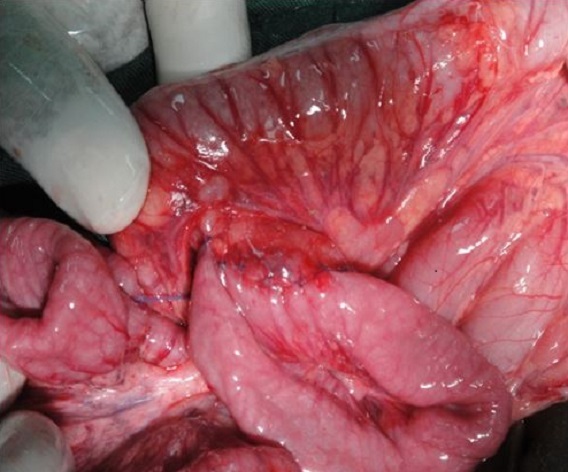
Vue opératoire de la duodéno-jéjunostomielatéro-latérale

Après un traitement médical associant un arrêt de l'alimentation orale, une aspiration digestive, une rééquilibration électrolytique et une correction de l'anémie, l'enfant a été traité chirurgicalement par une duodéno-jéjunostomielatéro-latérale sous mésocolique (Figure 4). Les suites étaient troublées au 4^éme^ jour postopératoire par l'apparition d'un pic fébrile à 39° et une rhinite purulente alors que l'examen de l'abdomen était sans particularité. Les hémocultures étaient positives à Enterobactersp sécrétrice de b-lactamase, sensible au chloramphénicol et à l'imipenème. Cette septicémie a été rapidement maitrisée par une antibiothérapie adaptée. Le transit gastroduodénal de contrôle au 9^éme^ jour postopératoire montrait un bon passage duodénal sans fuite au niveau de l'anastomose autorisant ainsi une alimentation orale. Le retour à domicile a eu lieu au 11^éme^ jour postopératoire. Les vomissements avaient disparu et l'enfant avait gagné 2 Kg avec un recul de 3 mois.

## Discussion

C'est à Rokitansky que revient la première description de syndrome de la pince aorto-mésentérique en 1861, Wilkie en 1927 a présenté la physiopathologie et le traitement à partir d'une série de 75 cas chez l'adulte [5]. Plusieurs noms lui sont prêtés : syndrome de l'artère mésentérique supérieure, cast syndrome (syndrome du corset plâtré), syndrome de Wilkie, iléus duodénal chronique etc. Il s'agit d'une affection rare, parfois méconnue et très peu de cas ont été publiés chez le nourrisson [3, 4]. Sur le plan physiopathologique, ce syndrome est lié à un espace aorto-mésentérique réduit, inférieur à 8 mm au niveau du troisième duodénum associé à un angle aorto-mésentérique inférieur à 20° [6, 7]. Normalement, l’épaisseur du tissu adipeux entourant l'AMS à son origine permet de maintenir cet angle ouvert et protège le duodénum de la compression vasculaire [8].

Plusieurs facteurs étiologiques sont décrits au premier rang desquels on retrouve un amaigrissement important ou une croissance staturale rapide sans prise de poids, à l'origine de la disparition de la graisse péri-mésentérique, favorisant ainsi le rapprochement de la pince aorto-mésentérique [1, 2, 7, 8]. La déformation du rachis lombaire (hyperlordose lombaire, déformations rachidiennes post-traumatiques ou post-chirurgicales) participe à ce mécanisme en projetant l'aorte vers l'avant [1, 3, 8]. Des facteurs anatomiques interviennent aussi dans la genèse de ce syndrome : l'insertion basse de l'AMS sur l'aorte, la brièveté du ligament de Treitz à l'origine d'une insertion haute de l'angle duodéno-jéjunal [1, 9].

Dans notre cas, aucune de ces anomalies n'a été retrouvée. Il s'agit vraisemblablement d'une forme primitive pouvant être en rapport avec des facteurs d'ordre génétique ou familial prédisposant à la survenue de l'affection d'autant plus que c'est un nourrisson issu d'un mariage consanguin du second degré. En effet, des observations familiales ont été décrites, faisant ainsi évoquer un facteur génétique [2]. Le syndrome de la pince aorto-mésentérique est un diagnostic qu'il faut savoir évoquer, devant des signes d'occlusion intestinale haute aigue ou chronique. Ainsi, la symptomatologie clinique se résume essentiellement à des vomissements répétés alimentaires ou bilieux [1, 4, 6, 10]. Les autres signes cliniques sont la distension épigastrique, l'amaigrissement et les troubles nutritionnels comme cela a été le cas chez notre nourrisson. Cependant, le diagnostic différentiel avec les autres causes d'occlusion duodénale ou un mégaduodénum peut être difficile [1]. Par conséquent, le diagnostic de certitude est apporté par l'imagerie médicale. Le transit gastroduodénal met en évidence des signes indirects à savoir une dilatation gastrique et duodénale avec arrêt linéaire incomplet du produit de contraste au niveau de D3 [3, 7]. La tomodensitométrie abdominale injectée est l'examen le plus performant pour le diagnostic. Elle retrouve une dilatation duodénale en amont de l'obstacle et permet de mesurer la distance entre l'aorte et l'AMS. Normalement, cet écart est compris entre 10 à 28 mm et l'angle aorto-mésentérique entre 45° et 60° alors qu'en cas de syndrome de la pince aorto-mésentérique, l'angle est fermé et ne mesure plus que 6 à 15° et la distance est de moins de 8 mm [6, 8, 10].

L’évolution est le plus souvent favorable lorsque la prise en charge thérapeutique est précoce et adéquate. En revanche, en cas d’évolution aigue, des complications à type de désordres hydro-électrolytiques, de troubles respiratoires et de perforation gastrique par dilatation aigue peuvent être fatales [1, 6, 7, 11]. Notre malade présentait la forme chronique avec comme complications des troubles hydro-électrolytiques. Le traitement est avant tout médical. Il repose sur la mise en place d'une sonde naso-gastrique laissée en aspiration douce, la correction des troubles hydro-électrolytiques et la nutrition parentérale ou entéro-jéjunale [2, 7]. Le traitement postural (décubitus latéral gauche, procubitus) n'améliore la symptomatologie que dans 50% des cas [1].

La chirurgie n'est indiquée qu'en cas d’échec du traitement médical. En réalité, elle s'avère nécessaire dans 75% des cas [7]. Plusieurs techniques chirurgicales ont été décrites: la section du ligament de Treitz, le repositionnement antérieur du duodénum, la translation rétro-mésentérique du duodéno-jéjunum, la duodéno-jéjunostomie [3, 6, 7]. La translation rétro-mésentérique du duodéno-jéjunum constitue le traitement de choix chez l´enfant; elle évite une anastomose digestive néanmoins elle n´empêche pas une éventuelle duodéno-jéjunostomie ultérieure en cas d´échec [1]. Le caractère chronique du tableau clinique, le long séjour hospitalier que nécessite le traitement médical, et les résultats aléatoires du traitement postural nous ont fait opter pour une cure chirurgicale. Notre choix est allé à la duodéno-jéjunostomie car elle procure les meilleurs résultats [6, 10, 11]. Elle peut être réalisée par laparotomie ou plus récemment par laparoscopie [12]. Les suites opératoires sont généralement favorables, marquées par une prise de poids et la disparition des vomissements comme l'illustre notre cas. La septicémie à Enterobactera été mis dans le cadre d'une infection nosocomiale.

## Conclusion

Le syndrome de la pince aorto-mésentérique est une affection rare chez le nourrisson. Les formes acquises sont les plus fréquentes mais il existe des formes congénitales. Il faut savoir y penser devant des vomissements bilieux et/ou alimentaires chez tout nourrisson qui présente un retard pondéral. Le diagnostic de certitude est basé sur le transit gastroduodénal et la tomodensitométrie injectée. Le traitement est d'abord médical mais dans les formes chroniques le traitement chirurgical s'avère nécessaire. La duodéno-jéjunostome par laparotomie ou par laparoscopie procure de bons résultats.
